# Supplementary material for: Are carbon emissions trading and green financial instruments synergistic? -Comprehensive quantitative research based on content analysis
Source: PLoS One. 2024 Mar 7;19(3):e0298601. doi: 10.1371/journal.pone.0298601 (PMC10919601; doi:10.1371/journal.pone.0298601)
Supplement: S1 Data — (ZIP) [file pone.0298601.s001.zip › Data/地级市Z_F工作报告.zip!/╜¡╬≈/╝¬░▓╩╨/└┤╫╘╣1⁄2╓┌║┼í╛┬φ┐╦╩2╛▌═°í┐_2015.docx]

各位代表：

　　现在，我代表市人民政府向大会报告工作，请予审议，并请各位市政协委员和列席会议的同志提出意见。

　　一、2014年工作回顾

　　2014年，在省委、省政府和市委的坚强领导下，全市上下认真贯彻落实省委“发展升级、小康提速、绿色崛起、实干兴赣”十六字方针，坚持“改革创新、扩大开放，提速升级、稳中求进”，统筹做好稳增长、促改革、调结构、惠民生等各项工作，较好完成了市三届人大四次会议确定的主要目标任务，奋力迈出了建设开放繁荣秀美幸福新吉安的坚实步伐。

　　—— —一年来，我们既保持定力，更主动发力，经济发展呈现稳中有进新气象。坚持把稳增长放在首位，全市经济运行逆势上扬、速度效益同步提升。总量稳步壮大。全市规模工业增加值688.6亿元、增长12.5%，限额以上消费品零售额增长23.7%，农业增加值增长5.4%，增幅均列全省第一；生产总值1242.1亿元、增长10.2%，社会消费品零售总额340.8亿元、增长13.5%，实际利用外资7.86亿美元、增长14.8%，增幅均列全省第二；引进省外资金447.8亿元、增长19.4%，外贸出口40.2亿美元、增长21%，增幅均列全省第三。质量持续提高。三次产业结构调优为16.78:51.13:32.09；战略性新兴产业和高新技术产业占规模工业比重达50.5%和27.3%，分别提高0.7和1.2个百分点；全市财政总收入195.2亿元，增长15%，占GDP比重提高0.6个百分点。要素支撑有力。固定资产投资1270.6亿元、增长19.4%；金融机构人民币各项贷款余额876.9亿元、增长22.7%，存贷比提高4个百分点，首次突破50%；工业用电量增长7.8%；货运周转量增长13%，全市经济活跃向好。

　　—— —一年来，我们既强化对接配套，更注重实践探索，改革创新展示砥砺奋进新气势。全面深化改革起步有力、蹄疾步稳，荣获全省全面深化改革优胜单位。改革路径明晰，按照市委确定的改革重点和主线，细化出台30多个配套文件，研究制定20多项重大规划，先后召开13场新闻发布会，营造了浓厚的改革氛围。改革领域广泛，既有吉泰走廊先行先试、井开区开放搞活等经济领域的改革创新，又有教育、卫生等社会事业的开放合作，还有政府的自我加压、简政放权，呈现真改、实改的良好态势。改革亮点纷呈，工业园区体制改革深入推进，财权事权统一的财政收入分成机制基本确立，园区发展活力明显增强；扩权强镇列入全省改革推广项目，吉州区“人文社区·温馨家园”创建获评中国社区治理十大创新成果；安福县省直管县改革扎实推进；吉安县职业教育、永丰县家庭农场、新干县公共文化服务、庐陵新区综合执法等改革各具特色、富有成效。

　　—— —一年来，我们既量力而行，更尽力而为，社会建设展现和谐共进新气场。始终把增进百姓福祉作为工作的出发点和落脚点。民生投入持续增多，全市民生支出243.3亿元，占财政总支出的78.6%。省市安排的60件民生实事全面完成。城镇居民人均可支配收入24797元、增长10.1%，农村居民人均可支配收入9262元、增长11.4%。社会事业持续进步，吉安职业技术学院顺利开学，填补了全市高等职业教育空白，上海东方医院吉安医院加快建设，全市首个院士工作站挂牌成立，成功承办第三届全国农民画展，荣获全国民族团结进步模范集体。对外影响持续扩大，全国电子信息百强企业发布会，以及全省镇村联动建设、商贸流通、现代农业示范园区建设、干部学用技术、公共文化服务等现场会相继在吉安召开；众多国家和省级主流媒体聚焦吉安、宣传吉安，吉安对外形象不断提升。

　　一年来，重点抓了以下工作：

　　（一）加快发展升级，产业支撑力继续增强。扎实推进四化同步互动、三产共生演进，产业结构不断优化。工业产业加快集聚。全市规模工业主营业务收入2809.6亿元、增长15.9%；规模以上企业达963户。战略性新兴产业实现主营业务收入1418.8亿元，尤其电子信息产业突破500亿元，占据全省“半壁江山”，成功获批国家新型工业化电子信息产业示范基地，航盛电子获“江西首届省长质量奖”提名奖，红板、合力泰跻身全国电子信息百强企业行列，合力泰成功上市，实现企业境内主板上市“零突破”。农业结构不断优化。粮食生产实现“十一连丰”，粮食收购量达42亿斤、居全省第一。富民产业不断壮大，井冈蜜柚“千村万户老乡工程”深入推进，新增面积11万亩、覆盖农户5.7万户；茶产业形成了种植加工到文化创 意全产业链；烟叶产量突破30万担，成为全国烟叶重点产区。农业产业化水平不断提升，新增家庭农场2407家、专业合作社979家，省级龙头企业达94家。现代农业示范园开放搞活，形成了以井冈山国家农业科技园为龙头、各县市区为支撑的“1+13”园区体系，逐步成为农民培训、农业升级、农旅结合的主战场。农业综合生产能力不断增强，完成水利建设投资19亿元，新建高标准农田16万亩。现代服务业扩量提质。商贸物流势头正旺，中心城区十大市场体系建设已见成效，天虹购物中心、城南生产资料专业市场等一批高端城市综合体建成营业，井开区综合物流园评为全国十大商贸物流信息平台，中心城区12个菜市场改造升级。电子商务方兴未艾，新增规模电商企业21家，实现交易额104.8亿元、增长131.7%。旅游业回暖升温，全市接待游客2928.6万人次、增长9.6%，实现旅游总收入226.3亿元、增长10.5%；青原山突破体制障碍，景区建设加快升级，阳明书院对外开放；武功山整体开发加快推进，羊狮慕景区基本建成；井冈山旅游标准化示范区建设通过国家验收，罗浮片区启动建设；中心城区古后河绿廊成为旅游新热点；全市新增国家4A级景区3个。金融业快速发展，实现税收超过9亿元、增长36.8%，成为全市发展的重要引擎。房地产业平稳健康发展，市中心城区商品房新开工面积增长17.8%。全市服务业实现税收85.5亿元，占财政总收入的43.8%。

　　（二）深化重点带动，板块聚集力继续增强。深入实施重点带动战略，推动区域优化升级。苏区振兴“红利”加快释放。积极加强与国家部委、省直单位的对接沟通，全市33个重大平台获国家和省里批复，6个重大平台列入省重点调度；吉水中电新材料成为全省苏区振兴对口支援首个落户的产业扶助项目。在苏区振兴战略的带动下，全市争取各类资金171亿元、增长10%，争取上级调剂用地指标1.8万亩，占全省的五分之一。井开区“龙头”加快昂起。完成基础设施投入61亿元，深圳大桥竣工通车，电子专业市场投入运营，1200套创业公寓建成投用。24个亿元以上项目进驻落户。全年实现规模工业主营业务收入460亿元，其中电子信息产业达200亿元，占全市的40%。吉泰走廊“脊梁”加快挺起。产业集聚度不断提升，工业主营业务收入占全市的57%；电子信息产业主导地位日益凸显，已聚集电子信息企业250多家，实现主营业务收入417亿元，占全市的83.4%。吉泰城镇群建设加快推进，105国道改道及永和连心大桥竣工通车，新井冈山大桥、庐陵大桥、吉水赣江二桥和恩江二桥加快建设，走廊快速通道和外环线全面贯通。水电油气、通讯交通、医保、金融和劳动力市场基本实现一体化，50%的乡镇开通公交。吉泰走廊生产总值占全市的51.2%，提高1.1个百分点；财政总收入迈上百亿台阶，达107.8亿元，占全市的55.3%，提高1.1个百分点，走廊支撑引领作用进一步增强。县域经济“支点”加快立起。一批县域特色产业快速壮大，井开区通讯终端设备、吉安县数字视听、永丰循环经济3个产业集群过百亿，永丰碳酸钙、吉水绿色食品产业集群过80亿元，井开区医药化工、吉州区装备制造和通讯传输、泰和触控显示、安福液压机电、新干盐卤药化6个产业集群过50亿元。8个县财政总收入迈上10亿元台阶，其中吉安县过22亿元。

　　（三）全面深化改革，发展内生力继续增强。紧扣“区域先行、市场先导、管理放权、机制放活”改革主线，重点推进“1+16”改革措施。吉泰走廊“六个一体化”先行先试有效实施，72项改革已完成30项，井开区人事制度改革、投融资机制创新取得重大突破；旅游景区经营体制有效放活，渼陂景区“三权分离”试点深入开展，市庐陵文化旅游公司全面运营；农村集体土地承包经营权确权登记全面推开，国有林场改革基本完成。电子政务协同办公系统实现市县乡“全覆盖”，市本级政府机构改革基本完成，行政审批和商事制度改革取得新成效，精简审批权467项，精简率达74%，大大激发了市场活力和创业热情，全市新增注册登记企业户数5930家、增长31%，注册资本额增长130.6%。金融体制创新迈出新步伐，交通银行和南昌银行吉安分行、泰和中银富登村镇银行开业运营，在全省率先成立民间借贷登记服务中心和民间资本管理公司。

　　（四）狠抓项目建设，投资拉动力继续增强。坚持把扩投入作为稳增长的关键举措，推动大开放、大投入、大发展。项目投入持续加大，392个重点项目扎实推进，一批水利、电力、交通等重大基础设施项目加快建设，峡江水利枢纽7台机组并网发电，昌吉赣客专、蒙华铁路煤运通道开工建设，吉安开启高铁新时代。招商引资势头强劲，先后在深圳、福州、宁波、苏州和港澳台等地举办专题推介活动10多次，新引进5000万元以上工业项目230个、农业项目37个、服务业项目56个，木林森、中广核等一批投资50亿元以上重大项目成功落户。对台招商再创佳绩，全市新增台资项目25个，实际利用台资1.5亿美元，引进台资项目数和利用台资总额均列全省第一。社会融资稳步扩大，全市融资285.29亿元，其中银行融资195.3亿元，企业债券融资45亿元，地方政府债券融资5.8亿元，企业上市融资2.79亿元，小额贷款公司融资36.4亿元。

　　（五）统筹城乡建设，环境承载力继续增强。坚持城乡统筹、镇村联动，持之以恒推进新型城镇化和美丽乡村建设，大气雅气秀气吉安的特色与魅力全面彰显。遵循“大规划、大贯通、大组团、大生态”的思路，城区建设多点发力、组团推进。中心城区195个重点城建项目顺利实施，赣西堤路网建设全面启动，市全民健身体育中心项目有序推进，十个总部大楼、六个金融大厦加快建设，庐陵新区总部经济街加快形成；金樟组团实现规划一体化，金樟大道基本贯通；青原山大道竣工通车，拉开了中心城区怀抱青原山发展新序幕；吉安县、吉水县融城步伐加快，泰和县五大城市综合体同步建设，新干与吉水滨江景观带、永新将军大道景观绿化成为靓丽风景线，遂川、永丰、万安高速连接线改造升级，大吉安、组团式发展格局更加清晰。同时，城市功能品位和管理水平加快提升，古后河绿廊、螺湖湾湿地公园加快建设，白鹭洲书院修缮复建，仓口庐陵古街改造进展顺利，庐陵文化生态保护实验区全面启动，城市人文气息更加浓郁；中心城区新增公交班次260个，公交满意度调查列全省第一；智慧城市建设有效推进，“数字吉安”试点通过验收，中心城区数字化城管进入试运行。全市城镇化率提高1.52个百分点，达到44.65%。落实“严格规划、带状推进、镇村联动、扩权强镇、产业升级、生态永续”的要求，彰显庐陵风格的美丽乡村建设扎实推进。高标准完成1003个村点建设，实施71个集镇、226个村庄联动建设，打造了一批特色鲜明、带动力强的示范镇村；耕地保护成效明显，农民建房“两违”现象有效遏制；全市新增4个中国历史文化名镇名村、8个中国传统村落、7个国家级“非遗”项目、2个中国少数民族特色村寨。

　　（六）推进生态建设，绿色竞争力继续增强。全面落实市人大《关于加强吉泰走廊生态保护的决议》，在全市范围实行生态红线管控，确立发布第一批67个市立公园，“精品公园＋生态绿廊＋郊野公园”城镇三级生态梯度格局更加完善。大力开展工业污染、农村面源污染等环保专项整治行动，空气环境质量稳定在国家Ⅱ级标准，赣江吉安段及支流地表水保持在国家Ⅲ类标准以上，城市饮用水源水质达标率100%，大中型水库水质达标率90%。万元GDP能耗下降3.3%，节能减排完成年度目标任务。“四城同创”取得显著成效，环境卫生、市民素质大幅提升，省级文明城市指数测评全省第一，成功创建国家卫生城市、国家森林城市和首届江西省生态宜居城市。生态建养扎实推进，新增绿化造林47.9万亩，新增国家级生态乡镇12个、省级生态乡镇村16个。

　　（七）强化民生保障，社会凝聚力继续增强。“千人创业万人就业工程”成效显现，新增小额担保贷款13.1亿元，扶持个人创业1万余人；新增城镇就业5.5万人、转移农村劳动力7.2万人。居民消费价格总水平涨幅2.1%，低于全省平均涨幅0.2个百分点。城镇登记失业率2.98%，低于全省平均水平0.29个百分点。基本建成保障性住房6956套，完成棚户区改造11354套，新开工保障性住房15300套、棚户区改造19711套，完成农村危旧土坯房改造1.3万户。农村低保扩面2.2万人，保障面提高0.5个百分点。连续十次提高养老金标准，全市退休人员人均养老金每月达1603元，增长15.5%。扶贫攻坚扎实有效，建成农村公路2290公里，23万农村人口喝上安全水，完成移民扶贫搬迁1.3万人，减少贫困人口9.1万人。科技创新加快推进，新增省级科技创新平台8个、省级产业技术创新战略联盟2个。城乡教育网点更加优化，吉师附小新校园和吉安一中新校区落成开学，白鹭洲中学新校区二期加快推进，全市恢复农村教学点54个，完成农村义务教育标准化建设和薄弱学校改造项目821个，新建乡镇中心幼儿园74所；“圆梦助学金”和“高考入学政府资助金”共资助贫困学生3500多名，高考录取率达83%，高于全省平均水平8.8个百分点。新农合大病保险在全省率先铺开，补偿人次、补偿金额均居全省第一，社区卫生服务实现全覆盖，新建医疗“空白村”卫生室122所，人口与计生工作荣获全省先进。公共文化服务标准化、均等化、社会化稳步推进，吉安国家印刷包装产业基地正式挂牌；全民健身活动深入开展。社会治理成效明显，食品药品监管和安全生产形势总体平稳，平安建设荣获全省先进，公众安全感居全省第二。同时，驻吉部队和民兵预备役人员积极服务地方发展，在急难险重任务中发挥重要作用，应急处突能力不断提升，国家安全、国防动员、拥军优属、人民防空、防震减灾等工作进一步加强，统计、供销、方志、档案、外事侨务等工作取得新成绩，妇女儿童、社会慈善、红十字会、残疾人及老龄事业实现新发展，气象、水文、海事、盐务、石油、电力、机场、海关、检验检疫、高速路政、邮政通信等中央和省属单位服务地方作出了新贡献。

　　（八）加强自身建设，政府公信力继续增强。扎实开展党的群众路线教育实践活动，先后启动拖欠农民工工资、安全生产等6个专项治理，开展民生资金使用管理、“三公”经费、精简会议文件和节庆论坛等16项专项整治，“三公”经费支出下降12.7%，一批影响群众利益的突出问题得到有效解决，市政府党组教育实践活动群众满意率达99.3%。深入推进依法行政，自觉接受市人大的法律监督和市政协的民主监督，认真听取人大代表和政协委员的意见建议，全年办理市人大代表建议151件、市政协提案180件，办结率、满意及基本满意率均为100%。严格贯彻中央“八项规定”，认真执行国务院“约法三章”，全面落实廉政建设责任制，加强行政权力运行监督，强化政府投资工程监管，深入开展农村集体“三资”审计。大力加强效能建设，服务意识不断增强，发展环境不断改善，得到广大群众和客商普遍认可。积极推进政府信息公开，认真办好行风热线，受理办复群众来信来电来访2.8万件次，努力为人民群众排忧解难。

　　各位代表！成绩来之不易。我们深知，吉安的每一步发展、每一个变化，都得益于省委、省政府和市委的正确领导，得益于市人大、市政协和社会各界的鼎力支持，得益于全市人民的共同努力。在此，我代表市人民政府，向广大干部群众，向历任老领导、离退休老同志，向各民主党派、工商联、无党派人士和社会团体，向驻吉人民解放军、武警官兵和公安消防干警，向苏区振兴11个挂点帮扶国家部委机关，向所有关心和支持吉安发展的同志们、朋友们，致以衷心的感谢和崇高的敬意!

　　各位代表！回顾过去一年，我们也清醒地认识到全市经济社会发展还存在一些困难和问题，主要是：综合实力仍然不强，产业规模不大、结构不优、层次不高的问题仍较突出；改革创新的力度、广度和深度还不够，市场化程度不高，创新驱动不足，体制机制障碍依然存在；部分群众生活还比较困难，与全国全省同步全面小康的任务还十分艰巨；政府职能转变和能力建设有待加强，少数部门创新意识、担当意识、法治意识和服务意识有待增强，个别公职人员违法乱纪、贪污腐化、精神懈怠，不作为、慢作为、乱作为现象仍有发生，基层“三资”管理有待加强，办事效率有待提升，政务环境仍需改善。对此，我们一定高度重视，并在今后工作中努力加以解决。

　　二、2015年工作安排

　　2015年是全面深化改革的关键之年，是全面推进依法治国的开局之年，也是“十二五”的收官之年。新的一年，以“中高速、优结构、新动力、多挑战”为主要特征的经济发展新常态，既给我们带来了经济下行压力加大、转型升级任务艰巨、要素制约日益趋紧等困难和挑战，同时也为我们带来了更多机遇，加快发展的有利条件仍然多于不利因素。世界经济转型调整，为我们扩大开放、承接产业提供了难得机遇。国家大力推进长江经济带、“一带一路”建设，深入实施苏区振兴发展、罗霄山片区扶贫攻坚、生态文明先行示范区等发展战略，为我们加强区域合作、争取项目资金，带来了多重叠加的政策机遇。省委、省政府高度重视吉安发展，明确把吉泰走廊作为全省“连南接北”的重要纽带，支持走廊打造成重要增长带。特别是经过多年的不懈努力，吉安的发展势头日益强劲，为下一步发展蓄积了能量、奠定了基础；全市上下风清气正、团结奋斗、合力共为的良好政治生态，为加快发展提供了重要保障。只要我们解放思想、勇于创新，坚定信心、保持定力，攻坚克难、敢于担当，就一定能够适应新常态、抓住新机遇、实现新作为，推动经济发展再上新水平、迈上新台阶。

　　2015年政府工作的总体要求是：深入贯彻落实党的十八大和十八届三中、四中全会、中央经济工作会议，以及省委十三届九次、十次全会、全省经济工作会议、市委三届九次全会精神，主动适应新常态，坚持稳中求进总基调，坚持做大总量与提高质量效益并重，继续实施四化同步互动、三产共生演进主战略，着力发展升级，着力改革开放，着力生态文明，着力法治建设，着力民生改善，始终保持定力，坚定信心，排难而进，为建设开放繁荣秀美幸福新吉安、与全国全省同步全面小康而努力。

　　今年全市经济社会发展的主要预期目标是：生产总值增长10%，规模工业增加值增长12%，农业增加值增长4%，服务业增加值增长10%，财政总收入增长11.5%；固定资产投资增长18.5%，社会消费品零售总额增长13.5%，外贸出口增长8%，实际利用外资增长12%；城镇居民人均可支配收入增长10%，农村居民人均可支配收入增长11.5%，居民消费价格总水平涨幅控制在3%以内，城镇登记失业率控制在3%以内，万元GDP能耗下降3%，节能减排完成省下达的计划任务。

　　工作中，我们将努力做到“五个坚定不移”：一是坚定不移把加快发展作为首要任务，主要经济指标增速保持略高于全省平均水平、略高于全面小康年度目标要求，努力实现追赶型发展；二是坚定不移提升发展质量，转变发展方式，调优经济结构，努力实现更有质量、更高效益、更可持续的发展；三是坚定不移推进改革创新，以解放思想为先导，坚持改革不停顿、开放不止步，向改革要红利，向开放要动力，向创新要效益；四是坚定不移走绿色崛起之路，加强生态文明建设，确保经济增长建立在提高效益、节约资源、保护生态的基础上；五是坚定不移强化民生保障，推进依法治市，维护公平正义，确保社会和谐稳定。

　　围绕上述目标和要求，重点抓好以下九个方面的工作：

　　（一）强化区域升级，在打造重点板块上谋求新突破。深入推进苏区振兴发展战略，坚定不移“昂起龙头、挺起脊梁、立起支点”，既加快打造核心增长极，又力促多点发力、协同共进。

　　着力提高井开区“集聚度”。举全市之力支持井开区“决战千亿、昂起龙头”，狠抓产城互动，壮大产业集群，着力打造电子信息等战略性新兴产业和现代服务业集聚区，力争园区主营业务收入突破600亿元，尽快进入全省十强园区。超前谋划扩区调区，拓展发展空间。加快“东区开发、西区优强”，东区启动建设医院、郊野公园和教育产业园，高起点推开金鸡湖新区建设，尽快成为城南组团的城市新区；西区重点打造全省领先的物流配送平台，推进出口加工区升级为综合保税区。

　　着力提高吉泰走廊“贡献度”。坚持把吉泰走廊作为吉安融入全省发展格局的重要平台和战略支点，着力在功能布局、产业集聚、要素整合、城市组团、开放引资、开发合作、机制改革、社会治理、民生保障等方面持续聚焦发力，力争走廊生产总值占比提高1-2个百分点。推进产业集聚，突出发展以电子信息为主导的战略性新兴产业，力争走廊区域规模工业增加值突破400亿元、主营业务收入突破1700亿元。推进吉泰城镇群建设，完成金樟组团、敦厚永和凤凰组团、澄江沿溪南溪组团的详规编制，启动轻轨系统规划编制；加快井开区、吉水县、吉安县融城步伐，推进新井冈山大桥、庐陵大桥、金樟大道、吉水“一堤两桥”建设，抓紧实施亚行贷款吉安城市综合交通项目；加快建设泰和赣江大桥；高标准推进走廊卫星镇、优先发展镇和镇村联动建设。推进公共服务和要素市场一体化，开通卫星镇、重点镇、重点村到工业园区、中心城区、旅游景区公交线路，启动华能电厂向走廊内园区集中供热工程，推进水电气同质同价。

　　着力提高县域经济“均衡度”。按照“找准支柱、做强支点、夯实支撑、优化支持”的思路，打好县域经济发展升级“攻坚战”。培育壮大县域产业集群，引导各县市区加快打造1-2个主导产业集群，全市新增吉水绿色食品、永丰碳酸钙2个百亿产业集群，力争吉州区通讯传输、新干盐卤药化2个产业集群过80亿元，峡江医药化工、万安电子线路板、永新皮制品等7个产业集群过50亿元。落实安福县省直管县改革政策，推动泰和撤县设市。推行县域差异化考核，促进各县市竞相发展、均衡发展。力争全市11个县市区财政总收入过10亿元，其中5个县过15亿元。

　　（二）强化改革攻坚，在释放改革红利上谋求新突破。大力开展“全面深化改革推进年”活动，按照更加规范、更加扎实、更加注重实效的要求，既稳步推进中央和省里统一部署的公车制度、户籍制度等改革，又扎实推进富有吉安特色的各项改革。

　　加快政府综合改革。全面推进规划体系兼容整合，推动“多规合一”试点，加快形成“一张图、一张表、一个平台、一套机制”的政府管理体制。推进“三单一网”改革，有序接入全省网上审批系统，实现所有事项全流程、并联式网上公开审批。继续推进简政放权，实施流程再造，稳步推行“一口受理、限时办理、规范办理、透明办理、网上办理”的审批服务机制，最大程度提高行政效能。深化财税体制改革，力争完成“营改增”任务，启动财政专项资金项目管理改革。提高政府管理信息化水平，完善“数字城管”，争取国家“智慧城市”试点，建设“智慧吉安”。

　　推进市场化改革。深化商事制度改革，全面实行“先照后证”登记制。扩大公共服务领域开放，重点在市政、教育、文化、体育和健康养老等领域推出一批重大项目向社会资本开放，加快推动46个向社会资本开放的省级示范项目签约建设，稳步推进环卫绿化、城市管理等29个政府购买公共服务试点项目。深化投融资体制改革，推动投融资平台转型发展、市场化改革，建立借用还一体化、投建管相分离、实体化运作模式；探索政府与社会资本合作PPP、项目收益债等投融资模式，激发社会投资活力。

　　深化重点领域改革。深入推进吉泰走廊“六个一体化”改革先行先试，探索建立走廊区域集中审批制度，实施招商、审批、代办一体化；做大做强吉泰走廊投资开发有限公司，打造集项目孵化、投资并购、融资上市于一体的“一站式”投融资服务平台，推进金融一体化。加大工业园区扩权提效力度，将规划审批、项目报批等权限委托或授权给园区管委会。加快农业农村改革，完成农村土地承包经营权确权登记颁证工作，抓好农村集体产权制度改革试点，建立市县乡联网办公的农村产权交易中心，促进土地有序流转；推进扩权强镇，逐步将吉泰走廊20个卫星镇纳入试点范围，增强乡镇经济发展内生动力。稳步推广旅游景区“三权分离”经营模式，积极筹建市旅游发展集团公司，建立统筹运作、统一规划、统一管理的赣江沿线旅游开发协作机制。

　　（三）强化创新驱动，在推动工业升级上谋求新突破。坚持把工业崛起作为“定海神针”，打好“三年决战三个千亿”攻坚战，力争到2017年，全市规模工业增加值突破千亿、电子信息产业主营业务收入突破千亿、井开区主营业务收入突破千亿。

　　突出创新发展。推动企业协同创新，深化与高等院校、科研院所合作，新组建4个以上省级科技创新平台，组建电子信息和生物医药2个产业技术创新战略联盟。建立新兴产业创业投资基金。完善科技创新公共服务平台，落实专利资助和奖励政策，促进科技成果转化。深入推进质量兴市战略，支持企业争创名优品牌。

　　聚焦电子信息产业。设立电子信息产业发展专职机构，健全市场体系和服务机制，引进检测中心进园区，设立产业投资基金。实施“百亿企业培育计划”，优选一批科技含量高、带动能力强、市场前景好的电子信息企业进行重点帮扶，力争用2-3年时间将红板、合力泰、木林森等骨干企业打造成百亿企业。深入开展“产业集群提升行动”，着力打造通讯终端及传输、计算机及外部设备、触控显示、LED照明、数字视听和电子线路板等6大百亿集群，力争电子信息产业主营业务收入突破600亿元。同时，统筹推动绿色食品、生物医药、先进装备制造、新能源、新材料等产业集群发展。力争全市战略性新兴产业主营业务收入突破1600亿元。

　　提升园区发展水平。坚持把工业园区作为城市新区来规划建设，促进土地集约、企业集聚、产业集群、宜居宜业。创新建园模式，推进深圳产业园、香港六星产业园、木林森高科技园和吉水军民结合产业园等特色园区建设。完善配套设施，新建公租房3000套以上，组建或引进20条以上零担物流配送专线，每个园区建成1个便民服务中心。确保全市工业园区主营业务收入达到2800亿元，新增泰和、青原、吉水3个工业园区过200亿元，总数达到8个；吉安高新区突破300亿元，争取升格为国家高新区，并力争3年突破500亿元。

　　（四）强化富民增收，在发展现代农业上谋求新突破。坚持把现代农业作为“压舱石”，以壮大富民特色产业、打响绿色有机品牌、发展农产品精深加工为着力点，大力推进农业现代化，促进农业增效、农民增收。

　　继续调优农业结构。强化粮食安全责任，加大优质稻推广力度，新建高标准农田13.5万亩，确保全市粮食播种面积稳定在960万亩以上。狠抓富民产业上规模。深入实施井冈蜜柚“千村万户老乡工程”，设立蜜柚产业发展基金，强化技术服务，推广桃溪柚、金沙柚等优良品种，重点种植区域县市区各建设一个政府主导的井冈蜜柚苗圃基地，全市新增井冈蜜柚种植面积13万亩；抓好24个标准化蔬菜基地建设，新增蔬菜2万亩；推进茶叶品牌整合，全市新增有机茶叶3万亩；烟叶稳定在11万亩以上；发展壮大林业特色产业，新增高产油茶5万亩、珍贵楠木6万亩、竹木花卉10万亩。巩固发展畜禽水产业，保障农产品市场供应。同时，夯实农业发展基础，完成190座小（二）型病险水库除险加固，完善农技、农机、气象、水文、防灾减灾服务体系。

　　创优生态农业品牌。大力发展绿色有机农业，推进农业标准化、清洁化、品牌化生产，推广种养结合的生态农业循环模式，加强农产品质量安全认证，推出一批吉安农产品“绿色品牌”、“有机品牌”，力争全市新增无公害、绿色、有机农产品45个。坚持把现代农业示范园作为发展生态农业的重要载体，推进井冈山国家农业科技园和8个已成型的县级示范园提升水平、发挥效应，5个尚未授牌的示范园尽快成型授牌；放活经营机制，年内每个县级示范园引进农业龙头企业2家以上；强化科技创新，大力发展一批高效农业、观光农业、精致农业与设施农业，进一步提升吉安农业的影响力和竞争力。

　　狠抓农产品精深加工。引进培育农产品加工企业，推进井冈蜜柚产业园、食品加工园建设，每个县市区规划建设1个绿色食品产业园，引进1个亿元以上农产品加工项目，推动一产“接二连三”。加大农产品电商平台建设，每个县市区选择1-2个品牌农产品进行电商模式试点，探索农产品销售流通新业态。大力培育新型农业经营主体，新增家庭农场500家、农民合作社200家、市级龙头企业20家。

　　深入推进扶贫攻坚。继续用好苏区振兴发展、罗霄山片区扶贫攻坚等政策，做好“十三五”扶贫调研，优化整合扶贫资源，实行精准扶贫，确保扶贫到村到户。加快解决偏远山区交通、电力、通讯、安全饮水等基础设施“最后一公里”问题，加大城市公交向农村延伸力度，加快农村危桥改造，年内新修农村公路2000公里，解决12.8万农村人口安全饮水问题，完成扶贫移民搬迁1万人以上，减少贫困人口7.2万人。

　　（五）强化消费拉动，在发展服务业上谋求新突破。顺应个性化、多样化消费趋势，大力发展生产性和生活性服务业，推动服务业扩量提质。

　　提升发展旅游业。深入实施“三山一江”旅游战略，全力打造井冈山、青原山、武功山三大旅游产业集群，培育环中心城区旅游圈。井冈山重点加快罗浮片区和龙潭大峡谷综合开发，力争上市、“申遗”取得实质性进展；青原山重点建设农禅基地、游客服务中心和行思禅院，提升景区整体品质；武功山加快文家游客服务中心、羊狮慕景区和武功峡景区建设，积极融入全省“大武功”开发格局；实施赣江水上旅游整体开发，推进井冈山、永新、遂川红色旅游一体化，打造红色旅游精品城市，形成山上山下全域联动的旅游新格局。大力发展乡村旅游，重点抓好17个国家乡村旅游扶贫重点村建设，打造十大乡村旅游示范点。提升旅游品牌，支持青原山与吉州窑联合争创国家5A级景区、国家风景名胜区，古后河绿廊、白鹭洲书院、燕坊古村、永新三湾争创国家4A级景区。

　　创新发展商贸物流业。坚持“城内大商场、城郊大市场、城外大物流、网上大电商”的思路，推进商贸物流提档升级。继续实施传统市场提升改造，加快高端城市综合体建设，确保中心城区十大市场体系80%以上的项目如期竣工。启动跨境电子商务和国际物流工作，推动电子商务进农村，支持新干建设国家农村电子商务示范县、井开区争创国家电子商务示范基地，打造新兴电商城市。完善物流体系，提升吉安综合物流中心信息平台功能，加快井开区物流园、河西综合物流园和8个县物流园建设，推动集装箱、冷链、城市配送及快递物流发展。

　　加快发展金融业。把金融业作为战略性产业来打造，力争全市金融机构贷款余额突破千亿元，存贷比提高3个百分点。完善金融体系，积极引进股份制和地方商业银行，加快上饶银行和华夏银行吉安分行筹建开业；推进农村信用社改制；支持各类金融机构发起设立村镇银行，实现村镇银行县域全覆盖；扶持小额贷款公司、民间资本管理公司、民间借贷登记服务中心等新型金融机构发展壮大，打造全省民间资本管理示范区。健全融资担保体系，每个县市区设立1家注册资本5000万元以上的政府控股融资担保机构。优化金融生态建设，建立健全企业信用评价体系。加强政银企对接，建立银行抽贷报告制度与问题企业银行贷款协调机制，引导金融机构加大对实体经济扶持。抓好资本市场融资，推动企业上市、挂牌、发债等直接融资。

　　同时，落实政策措施，合理引导消费需求，促进房地产市场平稳健康发展。大力发展文化创意产业，开发提升剪纸、书法、农民画、陶瓷等文化产品。统筹发展信息服务、教育培训等新型业态，培育更多的经济新亮点。

　　（六）强化城乡统筹，在提升城乡品质上谋求新突破。围绕“干干净净、漂漂亮亮、秩序井然、和谐宜居”目标，统筹推进新型城镇化与美丽乡村建设，打造宜居宜业宜游美丽家园，力争城镇化率提高1.7个百分点。

　　着力提升中心城区“首位度”。整合资源、集聚力量，推动中心城区经济实力、内涵品质、功能配套与管理水平走在全市前列，成为“首善之区”。进一步明确功能布局定位，高标准推进庐陵新区商业综合体、总部街区、金融集聚区建设，打造江西中西部最具潜质的“产城融合先导区”；支持吉州区、青原区做大做强，力争2-3年吉州区进入全市第一方阵，青原区进入全市中游水平。加快推进金樟、城北、城东、城西、城南和城中“六大组团”开发建设，金樟组团重点加快道路交通基础设施建设；城北组团以庐陵文化生态园为中心，加快吉州工业园向西拓展，打造城市生态文明示范区；城东组团重点推进吉安国际文化创意产业园建设，提升滨江片区，加快打造魅力新城区；城西组团重点策应高铁建设，逐步拉开路网框架，同步配建公共设施，尽快形成城市综合交通枢纽；城南组团重点加快规划编制，推进吉安县城北新区道路网架、禾埠桥路堤结合工程建设，加快井开区基础设施及配套建设；城中组团核心区重点完善公建设施，推进铜锣湾等商贸网点建设。进一步提升城市功能配套和管理水平，新建5个街头小游园，建设油气一体供应站、公共停车场，启动公共自行车系统和快速公交系统建设，优化公交线路，加密班次，畅通城区交通；理顺物业管理体制，提升物业管理水平；争创全国文明城市。

　　扎实开展“三改三清”环境提升攻坚行动。按照属地为主、市县区联动的责任分工，集中3年时间在全市城区范围大力实施以“改旧城、改旧村、改旧厂，清河流、清广告、清违建”为主要内容的“三改三清”攻坚行动，实现人居环境大提升。着力改旧城，重点对房屋破旧、设施落后、安全隐患多的老城区、棚户区，分类实施市场开发、综合整治与保护提升，每个县市区完成3-6个棚户区或无物业管理住宅小区改造；深入推进“人文社区·温馨家园”建设，全市三分之二以上的社区创建达标。着力改旧村，对城中村、城郊村和风景名胜区村庄，分别进行置换改造、整合规范和保护提升。着力改旧厂，实施成片改造、拆旧开发和保护整治，加快青原区梅林工业园退园还城步伐。着力清广告，规范户外广告管理，清理整治主干道、商业街区和小街小巷违规广告，清除视觉污染。着力清河流，抓好河流截污整治，推进污水管网建设，综合治理赣江、禾河等河道水系。着力清违建，重点拆除街道和住宅小区的违章建筑，同步实施美化、绿化和硬化。中心城区和井冈山市率先完成“三改三清”，其他各县积极跟进。

　　全面提升小城镇和美丽乡村建设水平。优化小城镇布局，重点打造昌吉赣铁路沿线城镇带，以及安福——吉安——永丰、安福——永新——井冈山——遂川、井冈山——泰和3个城镇轴，抓好国家、省、市重点示范镇和扩权强镇试点镇建设，打造一批旅游名镇、商贸大镇、工业重镇和农业强镇。推进美丽乡村建设，突出规划管控，深入整治农村“两违”建房，严控山体、水体开发。突出文化传承，加大历史文化名村和传统村落、传统民居保护力度，建设美丽宜居乡村，加快由“千村一面”向“各美其美”转变。突出沿线整治、带状推进、镇村联动，既整治一般自然村，又加快培育中心村，每个县打造1-2个精品示范村、1-2条精品示范带，全市70%以上的镇村联动点基本成型。突出环境改善，深化农村清洁工程，推广污水处理“生态疗法”，推行“户分类、村收集、镇转运、县处理”的垃圾处理模式，推进集镇和中心村垃圾收集、污水处理设施建设。突出农村文明，提高农民素质，推动由“物的新农村”向“人的新农村”转变。

　　（七）强化开放推动，在全面扩大开放上谋求新突破。坚持对内放活与对外开放并重、“引进来”与“走出去”并举、搭建平台与优化环境并行，加快推动开放升级。

　　强攻招商引资。继续推进专业招商和“十大将”招商，注重以商招商，加强协会商会等市场招商，力争年内引进省外资金515亿元，实际利用外资8.8亿美元。拓展招商区域，巩固长珠闽、延伸京津冀、对接韩欧美，特别是铆住对台招商，每个县市区在台湾开展一次以上专题推介活动，加强吉台两地农业交流合作，力争全市引进台资项目30个，实际利用台资2.5亿美元。强化全领域招商，大力引进电子商务、总部经济等新型业态项目，每个县市区引进2个以上较大规模的现代服务业和农业项目。推进招大引强，实施重大项目招商引资三年攻坚计划，推动“央企入吉”，力争引进投资50亿元以上项目实现新突破。

　　深化全民创业。宣传创业文化，降低创业门槛，落实扶持政策，营造良好的创业环境。鼓励返乡创业，加快创业孵化园等平台建设，着力培植本土企业。支持青年创业，在井开区建立“两岸青年创业孵化基地”，在每个工业园区建立大学生创业孵化基地，开展创业初期辅导培训。完善人才信息库，健全人才培养和激励机制，加大高层次人才引进和奖励力度，推动人才与产业对接，激发创新创业活力。

　　拓展国际市场。融入国家“一带一路”倡议，支持企业“走出去”承包工程、对外投资，带动劳务外派和对外贸易。加快电子口岸平台建设，改造提升铁路口岸作业区，积极对接上海、福建、广东等地自贸区，谋划井冈山机场通关开放，争取开通南昌——吉安——深圳铁海联运“五定”班列。优化出口结构，完善重点出口企业联系制度，提高生产型企业自营出口和高新技术产品出口比重。

　　提升开放优势。加快交通和能源等重大基础设施建设，初步安排401个市县重点项目，总投资2403亿元，年度计划投资740亿元。年内实现峡江水利枢纽工程、江西成品油管道二期工程全面竣工投用，井冈山机场二期扩建、新干航电枢纽等项目开工建设。支持配合跨区域项目建设，实现南昌—— — 宁都高速公路建成通车，加快东乡—— — 昌傅高速公路、广昌—— — 吉安高速公路等项目建设，全力支持昌吉赣客专、蒙华铁路煤运通道建设。推进国省道提升改造。启动“十三五”规划编制，突出抓好战略谋划、要素支撑和项目储备。继续打好苏区振兴牌，紧盯国家“七大工程包”积极争资争项，加快推进重庆—— — 长沙—— — 厦门、吉安—— — 抚州—— — 武夷山、井冈山—— — 赣州、吉安—— — 永安—— — 泉州铁路，以及神华吉安火电厂、华电峡江火电厂、华能井冈山电厂三期扩建工程等项目前期工作。

　　（八）强化生态建养，在打造综合优势上谋求新突破。积极融入全省生态文明先行示范区建设，争创全国生态保护与建设示范区，着力打造“水清、河畅、天蓝、气净、路通、景美”的美丽新吉安。

　　全面推进生态创建。启动赣江两岸风光带、都市田园休闲观光带和吉安都市田园休闲观光区“两带一区”建设，力争“一年打基础、两年出形象、三年见成效”。沿赣江神岗山至吉水桃花岛，全面实施赣江沿岸立面整治、景观建设，形成亲水型、人文型滨水生态景观，打造人文历史与自然生态融合的风光带；沿青原山至东固公路，大力发展农家乐、乡村休闲游、农业体验观光游，打造绿色生态和庐陵古韵相辅相成的都市田园休闲观光带；在吉州兴桥区域，搞好规划管控，高标准建设美丽乡村与市区农业示范园，提升钓源古村景区，凸显庐陵建筑风格，努力打造集农业示范、休闲观光、乡村旅游于一体的都市田园休闲观光区。引导各县市对重要道路、河流沿线进行整体规划建设，打造一批特色风光带。深入实施生态家园创建工程，巩固森林城市创建成果，推进县市区三级生态梯度建设，确保全市生态乡镇达到60%。申报创建庐陵赣江和遂川五斗江国家湿地公园。

　　加大低产低效林改造力度，新增造林36万亩。加大环境保护力度。科学划定生态红线，将饮用水源保护区、风景名胜区、基本农田等划为禁建区，实行红线管控。严格执行环保“三同时”、生态环境损害“一票否决”与责任终身追究制度，严格落实水资源管理“三条红线”。深入开展“净空、净水、净土”行动，突出工业废气、机动车尾气和扬尘污染专项整治，强化中心城区PM2.5污染源头控制与监测；加强中小河流与水库水质的保护治理，狠抓畜禽养殖等农村面源污染防治和土壤重金属污染治理。开展噪音污染整治，打造“宁静城市”。

　　促进资源集约利用。严格落实节能减排责任制，继续抓好“六厂一车”等重点领域污染减排。大力发展循环经济，申报创建国家循环经济示范城市，推动循环经济产业园“城市矿产”示范基地建设，加快井开区和永丰省级园区循环化改造，争取井开区列入国家园区循环化改造示范试点。严守耕地保护红线，严格执行土地使用标准和投资强度要求，盘活城区、园区存量土地，提高土地利用率。

　　（九）强化民生改善，在增进百姓福祉上谋求新突破。始终坚持民生为本、民生优先，让发展成果更多惠及广大群众。

　　大力发展民生事业。落实省50件民生实事。扶持就业创业，推进“千人创业万人就业”工程，深入开展农民工职业技能提升和维权行动，确保新增城镇就业5.5万人，新增转移农村劳动力5万人。提升社会保障水平，将城市低保月人均保障标准提高到450元，农村低保标准提高到240元，农村五保户集中供养标准提高到305元；抓好机关事业单位养老保险“并轨”改革，启动城乡居民基本医疗保险制度整合；提升城乡居民大病保险保障水平，加大贫困患者重大疾病救治力度；完善养老服务体系，改造74所农村敬老院，新建50个城镇社区和200个农村居家养老点；新建保障性住房8440套，新开工棚户区改造13655户，继续抓好农村危旧房改造。加强食品药品生产、流通、消费全过程监管，创建全国食品药品安全示范区。狠抓安全生产工作，加强突发公共事件预警信息发布，提升应急处置水平，保障人民群众生命财产安全。

　　统筹推进社会事业。优先发展教育事业，改善农村办学条件，继续恢复一批农村义务教育教学点，完成农村义务教育学校标准化建设和薄弱学校改造项目700个，招聘农村教师1000名；大力发展职业教育，探索建立以吉安职业技术学院为龙头、各县市区中职学校有效对接、相关企业主动融入的职教联盟，加快金樟职教园区和吉安县华忆职教园区建设；加快普及学前教育，推进一乡（镇）一所中心幼儿园建设，力争年内50%以上乡镇建有中心幼儿园；优化中心城区教育布局，推动优质教育资源向新城区延伸；深入开展“圆梦助学”等济困活动，不让每一个孩子因贫困而失学。大力发展卫生计生事业。加快市医疗急救指挥中心、儿童医院等项目建设，支持市中心医院和井大附属医院加快升级，实现上海东方医院吉安医院顺利运营；推进县级公立医院综合改革，支持社会办医；优化整合计划生育利导政策，促进人口长期均衡发展。繁荣文化体育事业。全面推进基本公共文化服务标准化、均等化，实现市工人文化宫、城市展示馆、科技馆早日投用；加快创建国家级庐陵文化生态保护实验区，办好江西省第二届畲族文化艺术节；完善公共体育服务体系，加快市全民健身体育中心和社区运动场所建设；践行社会主义核心价值观，推动公共慈善事业发展，开展志愿者公益活动，倡导文明新风尚。做好新形势下民族宗教工作。加强国防动员和后备力量建设，推进重要领域军民深度融合，扎实开展新一轮全省全国“双拥”模范城创建工作。

　　深入推进法治吉安建设。坚定不移走中国特色社会主义法治道路，尊重宪法和法律权威，推进依法治市，以法治引领推动改革发展。依法保障社会公平，建立“法律顾问”制度，开展法律援助，加快健全以权利公平、机会公平、规则公平为主要内容的社会公平制度体系。依法推进诚信建设，建立守法诚信“红名单”、违法失信“黑名单”制度和市场禁入、退出机制。依法维护社会秩序，构建人民调解、行政调解和司法调解联动平台，落实“诉访分离”和“信访终结”制度，引导逐级信访，健全依靠法治、依靠基层、依靠群众预防化解矛盾的工作机制；加强社会治安综合治理，依法打击违法犯罪活动。抓好法治宣传教育，开展国家宪法日宣传活动；加强各级干部法律知识培训，提高运用法治深化改革、推动发展、维护稳定的能力，大力营造办事依法、遇事找法、解决问题用法、化解矛盾靠法的法治环境。

　　三、进一步加强政府自身建设

　　适应新常态、决战同步全面小康，对政府工作提出了更高要求。我们将切实加强政府自身建设，不断提升工作水平，尽心竭力为人民服务、对人民负责、让人民满意。

　　坚持依法行政，努力建设权责法定的法治政府。健全政府工作制度体系，促进政府机构、权限、程序和责任法定化，切实做到“法定职责必须为、法无授权不可为”。完善行政决策机制，落实公众参与、专家论证、风险评估、合法性审查、集体讨论决定的法定程序，确保行政决策的民主性、科学性与法定性。严格规范行政执法行为，推动综合执法，健全执法问责机制。提高依法治理水平，注重运用法治思维及方式研究问题、化解矛盾。大力支持市人大立法试点，认真执行市人大及其常委会的决定和决议，主动听取人民政协的意见和建议，认真办理人大代表建议和政协提案，自觉接受人大法律监督和政协民主监督，高度重视舆论监督和社会监督，确保始终在法治轨道上行使权力、履行职责。

　　始终牢记宗旨，努力建设情系百姓的为民政府。巩固党的群众路线教育实践活动成果，站稳群众立场，践行群众路线，像抓经济建设一样抓民生保障，像落实发展指标一样落实民生任务。更加注重体察民情，倾听群众呼声，了解群众意愿，做到重大决策为民而定、政策举措应民而出、安危冷暖唯民所系。更加注重保障民生，大力解决群众最关心、最迫切的问题，真正让群众得到实惠。更加注重纾解民忧，认真办理市长热线、市长信箱、政务微博等群众来信来访。更加注重汇聚民智，完善社会公示和听证制度，抓好政府信息公开和新闻发布，切实保障公民知情权、表达权、参与权和监督权。

　　保持实干定力，努力建设勤政高效的务实政府。以“钉钉子”的精神狠抓落实，一张蓝图绘到底。善谋“实干”之策，察实情、谋实招，沉静务实，力求目标切实可行、举措务实管用、成效经得起检验。大兴“苦干”之风，勤勉敬业、夙夜在公，勇于担当、敢于负责，久久为功、善做善成。力求“快干”之效，完善重大项目领导挂点、重点工作现场办公、重要事项督促检查制 度，全面提升工作效率；加强政府绩效管理，强化行政效能监察，努力形成政令通、节奏快、效率高的良好局面。

　　坚持从严治政，努力建设风清气正的廉洁政府。坚决贯彻中央“八项规定”，严格落实党风廉政建设“一岗双责”。深化源头治理，推进惩治和预防腐败体系建设，完善重点领域、关键岗位、重大审批等监管制度，把权力关进制度“笼子”，有效预防腐败。自觉践行“三严三实”要求，严格执行国务院“约法三章”，严格落实中办、国办“十个严禁”，严格控制“三公”经费支出，坚决防止“四风”反弹。严把监督关口，加强对工程招投标、土地出让、产权交易、政府采购等重点领域的行政监察和审计监督，加强对政府项目资金监督管理，以“零容忍”态度严查贪污腐败和各类损害群众利益的违法违纪行为，树立政府清廉、干部清正良好形象。

　　各位代表！新的一年孕育新的希望，新的征途承载新的梦想。让我们更加紧密地团结在以习近平同志为总书记的党中央周围，在市委的坚强领导下，高举改革大旗，凝聚发展力量，迈出坚实步伐，为建设开放繁荣秀美幸福新吉安、决战同步全面小康而不懈奋斗!
